# Toxicity Testing of Pristine and Aged Silver Nanoparticles in Real Wastewaters Using Bioluminescent *Pseudomonas putida*

**DOI:** 10.3390/nano6030049

**Published:** 2016-03-11

**Authors:** Florian Mallevre, Camille Alba, Craig Milne, Simon Gillespie, Teresa F. Fernandes, Thomas J. Aspray

**Affiliations:** 1 School of Life Sciences, NanoSafety Research Group, Heriot-Watt University, Edinburgh EH14 4AS, UK; fm203@hw.ac.uk (F.M.); t.fernandes@hw.ac.uk (T.F.F.); 2 Sciences and Technologies, Lille 1 University, Villeneuve d’Ascq 59650, France; camille.alba@etudiant.univ-lille1.fr; 3 Scottish Water, Juniper House, Heriot-Watt Research Park, Edinburgh EH14 4AP, UK; craig.milne@scottishwater.co.uk (C.M.); simon.gillespie@scottishwater.co.uk (S.G.)

**Keywords:** nanoparticle, ecotoxicity, aging, bioluminescence, bacterial bioreporter, wastewater

## Abstract

Impact of aging on nanoparticle toxicity in real matrices is scarcely investigated due to a lack of suitable methodologies. Herein, the toxicity of pristine and aged silver nanoparticles (Ag NPs) to a bioluminescent *Pseudomonas putida* bioreporter was measured in spiked crude and final wastewater samples (CWs and FWs, respectively) collected from four wastewater treatment plants (WWTPs). Results showed lower toxicity of pristine Ag NPs in CWs than in FWs. The effect of the matrix on the eventual Ag NP toxicity was related to multiple physico-chemical parameters (biological oxygen demand (BOD), chemical oxygen demand (COD), total suspended solids (TSS) pH, ammonia, sulfide and chloride) based on a multivariate analysis. However, no collection site effect was concluded. Aged Ag NPs (up to eight weeks) were found less toxic than pristine Ag NPs in CWs; evident increased aggregation and decreased dissolution were associated with aging. However, Ag NPs exhibited consistent toxicity in FWs despite aging; comparable results were obtained in artificial wastewater (AW) simulating effluent. The study demonstrates the potency of performing nanoparticle acute toxicity testing in real and complex matrices such as wastewaters using relevant bacterial bioreporters.

## 1. Introduction

The interest in engineered nanoparticles (NPs, *i.e*., materials with all three external dimensions <100 nm) presenting new or improved physico-chemical properties compared to bulk materials is clear [1,2]. Since the widespread application of products of nanotechnology the general knowledge about the toxicity of various NPs to a range of environmental model microorganisms has grown dramatically [3] although specific knowledge relating to NP transformations and fate in different environments, and related exposure and hazard, are still missing. Reports on the actual environmental release of NPs are multiplying [4,5,6] raising the concern on their potential adverse effects to the local microbiota [7]. As reported in the related scientific literature [8,9,10], the current assessment of NP toxicity in real matrices (e.g., wastewaters) remains limited in part due to a lack of suitable methodologies for testing [11].

The use of bacteria as environmental bioreporters has shown to be a suitable approach in ecotoxicology for applications with real matrices [12]. The development of bespoke genetically modified bioreporters (GMB) using environmentally relevant model bacteria (e.g., *Pseudomonas putida* (*P. putida*), *Bacillus subtilis*), directly extracted from matrices of interest [13], extends the scope of possibilities already demonstrated by numerous *Escherichia coli* based GMB reports [14]. Furthermore, since bacteria offer clear advantages over other environmental models for performing high throughput toxicity screening of NPs [15] and their use in nanoecotoxicology is already well documented [16,17]. However, the use of, specifically, bespoke GMB remains scarce for NP testing [18,19,20] and examples of applications using real matrices (e.g., wastewaters) are not reported yet. There are consequently underexploited avenues using bacterial GMB in nanoecotoxicology [21].

Sanchez *et al.* (2011) [22] have stressed that while the developments on the use of NPs for environmental remediation in polluted soils and waters were expanding, the corresponding information on the possible long-term effects on the microbiota were barely reported. Studies on chronic effects [23] and speciation of NPs are emerging [24,25,26,27]. However, the actual nanosafety testing using aged NPs in real matrices is hardly carried out, whereas the potential adverse effects of released NPs in the environment are more associated with aged than pristine NPs. There are consequently very pertinent gaps in the knowledge of environmental NPs fate and effects, especially considering the potential impacts of aging in real matrices.

We therefore tested the suitability of a bespoke bioluminescent *P. putida* GMB [18,28], originally isolated from activated sludge, for performing real-time toxicity testing of pristine silver (Ag) NPs using spiked real wastewater samples. Both crude (*i.e.*, CWs, non-treated) and final (*i.e.*, FWs, treated) urban wastewater samples from different wastewater treatment plants (WWTPs) were used. The effect of the matrix of exposure (e.g., biological oxygen demand (BOD), chemical oxygen demand (COD), total suspended solids (TSS), ammonia, chloride, sulfide, and pH) to the eventual toxicity of the spiked NPs was evaluated using a canonical correlation multivariate analysis and discussed alongside the NP characterization related information. The impact of aging on Ag NP toxicity to *P. putida* was, in addition, investigated in CW and FW matrices herein.

## 2. Results

### 2.1. Acute Testing Using Freshly Added Ag NPs

**Figure 1:**
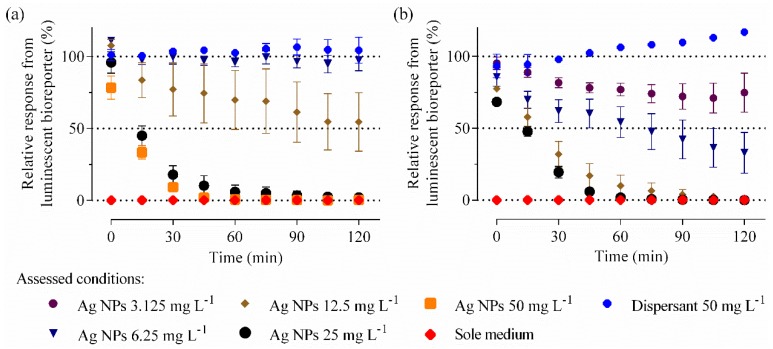
Real time monitoring of silver nanoparticle (Ag NP) toxicity in wastewaters. Relative luminescence output evolutions over time by *Pseudomonas putida* (*P. putida*) BS566::luxCDABE when challenged up to 200 mg·L^−1^ of Ag NPs in (**a**) crude and (**b**) final wastewaters from Site 2 are shown. Four out of the nine used NP concentrations are plotted; the entire graphics as well as results with samples from other sites are presented in Supplementary Materials (Figures S1–S4). Background signal from used matrices and effect of Ag dispersant (at 50 mg·L^−1^) are also presented. Data are mean ± standard error of the mean (SEM) (*n* = 4).

The ecotoxicity of pristine Ag NPs was assessed in CWs and FWs from four distinct WWTPs using the switch-off *P. putida* BS566::luxCDABE bioreporter. Light output evolutions over time obtained with spiked samples from Site 2 are shown in Figure 1.

A dose–response toxicity pattern characterized by a decrease in the signal output with increasing concentrations of Ag NPs was observed in both CW2 and FW2 samples. Greatest variability between replicated experiments occurred at 12.5 mg·L^−1^ of Ag NPs in CW2 and between 3.125 mg·L^−1^ and 6.25 mg·L^−1^ of Ag NPs in FW2, lower doses being non-toxic and higher doses being fully lethal at 1 h, respectively (Figure S1). Similar dose-response toxicity patterns were obtained for the other three site samples (Figures S2–S4). Crude samples, especially CW1 and CW4, showed the highest variability in terms of toxicity across experiments. Overall, Ag NPs were more toxic in FW than in CW samples.

Since Ag NPs were provided by the manufacturer as a 10% (w/v) suspension, the dispersant they are suspended in was tested separately. Results with the sole dispersant (tested at 50 mg·L^−1^, *ca.* ten times the derived IC_50_ (half maximal inhibitory concentrations) of Ag NPs) did not indicate antibacterial effect (Figures S1–S4). No background noise (*i.e.*, luminescence) was registered from the wastewaters *per se* in the absence of the bacterial bioreporter. Furthermore, in no case did the NP addition to the wastewaters resulted in increased output signal, regardless of the presence or absence of the bacterial bioreporter.

**Figure 2:**
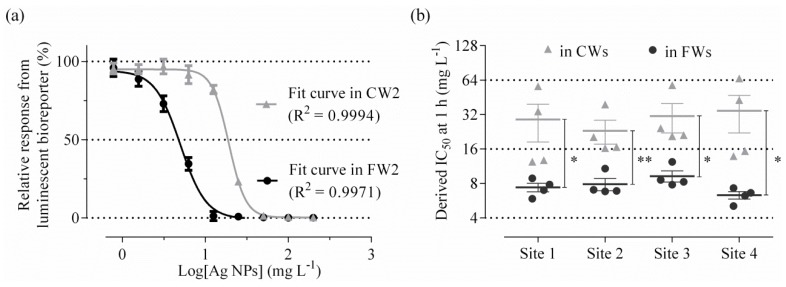
Derived toxicity values. Toxicity results were plotted as (response) = *f*(log[Ag NPs]) for selected time points and IC_50_ values (half maximal inhibitory concentrations) were derived by fitting a four parameter concentration–response model. An example of obtained fits for one test with crude and final wastewaters (CWs and FWs, respectively) from Site 2 (including four replicates *per* condition) is shown in (**a**). The comparison of all calculated IC_50_ values at 1 h is shown in (**b**). Data are mean ± SEM (*n* = 4), significant differences are represented by with *p* < 0.1 (*) or *p* < 0.05 (**) following analysis with unpaired t-tests. Derived IC_50_ values at 0.5 and 2 h are presented in Supplementary Materials (Figure S5).

Derived IC_50_ values at 1 h along with an example of generated fit curves for both types of wastewater are presented in Figure 2. All data were derived from good fits considering nine doses and exhibiting an average *R*^2^ of 0.9960 ± 0.0036 amongst all treated data (Figure 2a). Non-significantly different IC_50_ values of 7.4 ± 0.6 mg·L^−1^, 7.9 ± 1 mg·L^−1^, 9.3 ± 1 mg·L^−1^ and 6.3 ± 0.5 mg·L^−1^ were calculated in FWs for Sites 1 to 4, respectively (Figure 2b). The mean relative standard error (RSE) of all derived IC_50_ was inferior to 10%. By comparison, in CWs the mean RSE was close to 30% and the IC_50_ values derived were between 23.1 ± 5.4 mg·L^−1^ and 34 ± 12.5 mg·L^−1^ with no significant differences found between sites (Figure 2b). However, Ag NPs were found in all cases to have significantly lower toxicity in CWs than FWs. Comparable patterns were obtained at 0.5 and 2 h (Figure S5).

### 2.2. Wastewater Characterisation and Multivariate Analysis

A suite of relevant physico-chemical parameters including BOD, COD, BOD/COD ratio, TSS, pH, ammonia, chloride, sulfide and silver contents was considered alongside toxicity. Total plate counts were also used as a basic indicator of indigenous bacterial population size. Average data for all CW and FW samples from the different sites are presented in Table 1.

CWs showed significantly higher BOD/COD ratios (from 0.4 to 0.46) than FWs (inferior to 0.18 for FW1, 2 and 3 and close to 0.28 for FW3). The concentration of TSS was generally found at least ten times higher in CWs than in FWs and rather consistent between sites. A similar pattern was observed for the ammonia load, except for FW samples from Site 3. On an individual site basis, the pH was similar between samples and globally between 6.5 and 7, except for samples from Site 3 ,which exhibited one unit of pH difference between CWs (at 6.45 ± 0.09) and FWs (at 7.56 ± 0.06).

The original amount of silver was found below the lower detection limit of the AAS apparatus (<0.1 mg·L^−1^) in all samples. The total plate counts showed there were about 10^3^–10^4^ CFU·mL^−1^ in FWs and 10^6^–10^7^ CFU·mL^−1^ in CWs, regardless of the site. Comparatively to the other WWTPs, Site 3 presented a particular pattern with especially significantly higher amount of chloride and sulfide for CWs and significantly higher amount of chloride, ammonia as well as higher BOD/COD ratio, in addition to different pH readings for FWs. Samples from Sites 1, 2 and 4 appeared generally comparable.

**Figure 3:**
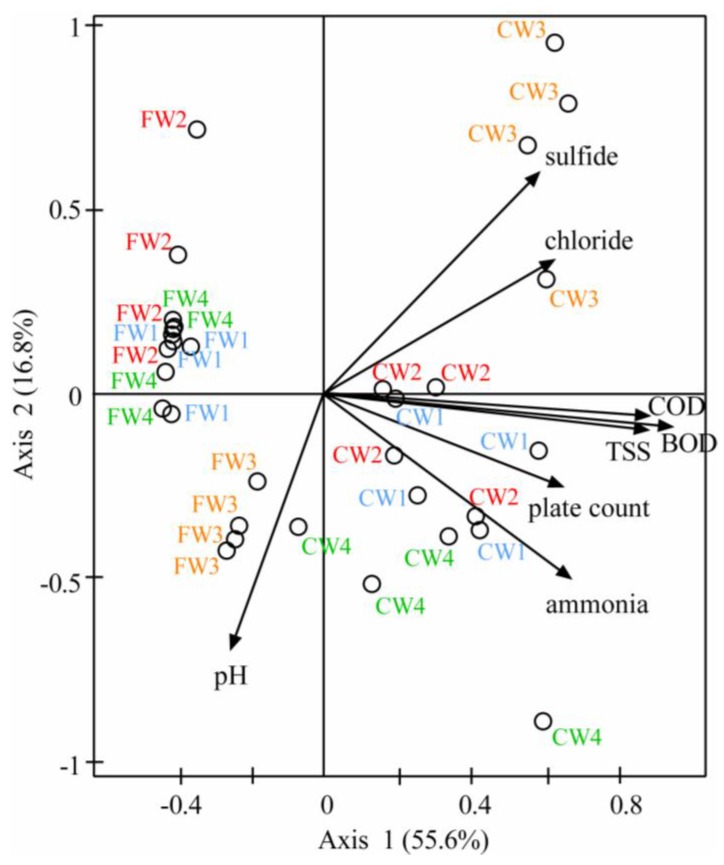
Results from multivariate analysis. Ordination diagram of thirty two wastewater samples from four wastewater treatment plants (WWTPs) (Site 1 in blue, Site 2 in red, Site 3 in orange and Site 4 in green) obtained by canonical correlation analysis considering height biochemical parameters (BOD, COD, TSS, ammonia, pH, chloride, sulfide and total plate count) as environmental variables constrained by one explanatory variable, the derived IC_50_ values at 1 h.

Results from the multivariate analysis carried out with the aforementioned environmental parameters constrained with the derived IC_50_ values at 1 h are presented in Figure 3.

Overall, 72.4% of the total variability was addressed by the two-way representation. Samples were clearly separated in FW and CW groups along axis 1 by mainly the BOD, COD and TSS parameters, which were highly related. In addition, sulfide, chloride, total plate count and ammonia information, supported this separation along both axes 1 and 2; these were also closely related. The highest variability was associated with the CWs as they were represented as generally more spread out than the FWs. Both FW3 and CW3 samples occurred isolated from the other FWs and CWs, respectively, and were mainly characterized by different sulfide, chloride and pH related data, as previously mentioned. The analysis with derived IC_50_ values at 0.5 and 2 h led to comparable results as global toxicity patterns were similar at those time points (data not shown).

### 2.3. Freshly Added Ag NP Characterization

**Figure 4:**
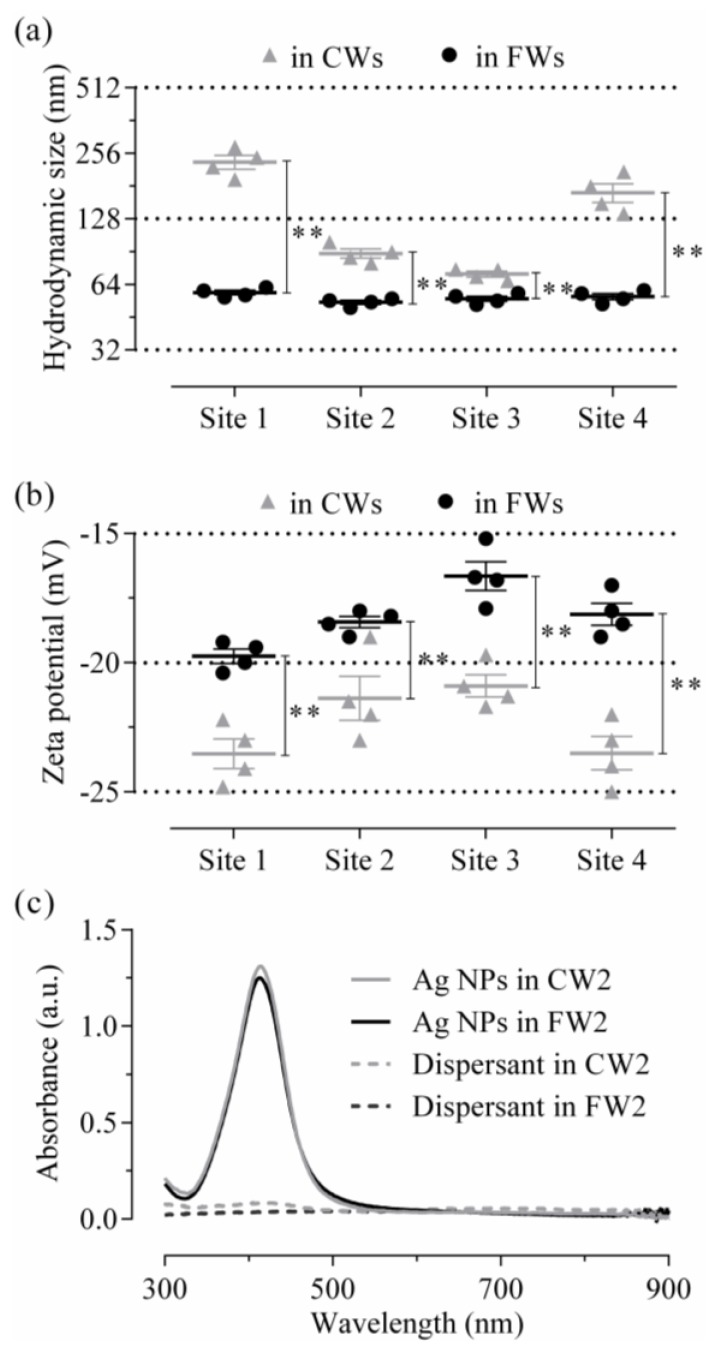
Ag NP characterization in wastewaters. Ag NPs at 10 mg·L^−1^ in crude and final wastewaters (CWs and FWs, respectively) were characterized by dynamic light scattering (DLS) and ultraviolet-visible spectroscopy (UV-vis). The hydrodynamic size results are shown in (**a**). The zeta potential data are plotted in (**b**). Data are in both cases mean ± SEM (*n* = 4), significantly different by unpaired t-test with *p* < 0.05 (**). In (**c**) is shown an example of typical spectra of absorbance (between 300 and 900 nm) obtained for spiked FWs and CWs from Site 2.

Ag NPs were characterized in wastewaters by dynamic light scattering (DLS) and ultraviolet-visible spectroscopy (UV-vis). Corresponding hydrodynamic sizes, zeta potentials and absorbance spectra are presented in Figure 4. Ag NPs showed consistent hydrodynamic size and zeta potential values in FWs between 53 ± 2.1 nm and 58.7 ± 2.7 nm and between −16.6 ± 1.1 mV and −19.7 ± 0.5 mV, respectively. In CWs, consistent zeta potential values were obtained between −20.9 ± 0.9 mV and −23.5 ± 1.1 mV, whereas hydrodynamic size varied between 71.3 ± 2.1 nm and 233.3 ± 16.9 nm. Hydrodynamic size and zeta potential values were both found significantly different between FWs and CWs, regardless of the site. All polydispersity index (PDI) values were between 0.4 and 0.6. Examples of the size distribution curves obtained by DLS (in intensity terms) are shown in Supplementary Materials (Figure S6). Registered spectra of absorbance were found similar between all samples and characterized by a single peak at 413.2 ± 0.8 nm in FWs and at 413.5 ± 1 nm in CWs for an average absorbance of 1.39 ± 0.12 a.u. (absorbance units) and 1.40 ± 0.2 a.u., respectively. Comparable low dissolution rates *ca.* 3% (regarding to mass) were equally obtained in both CWs and FWs at 1 h.

### 2.4. Impact of Aging

**Figure 5:**
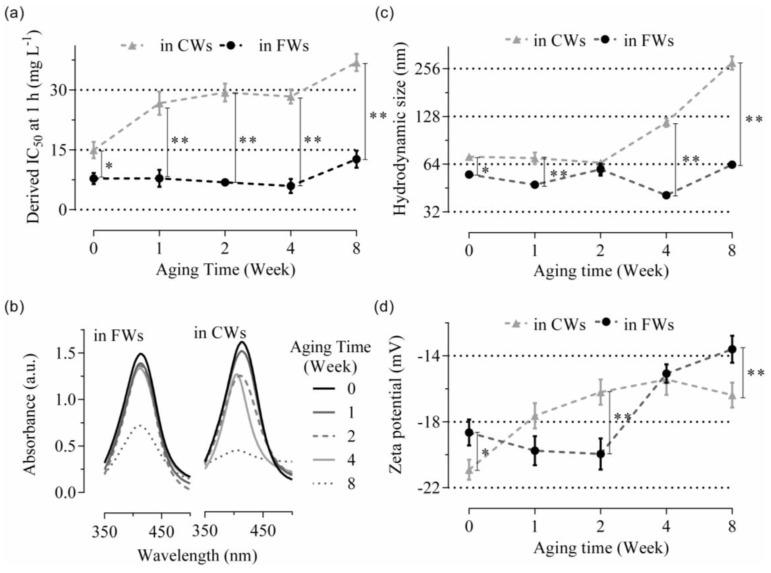
Effects of aging. Fate and toxicity of Ag NPs were tested after 0, 1, 2, 4 and 8 weeks of aging in FW and CW from Site 3. Derived IC_50_ values at 1 h from ecotoxicity assays are presented in (**a**). Absorbance spectra obtained by UV-vis with Ag NPs at 10 mg·L^−1^ are presented in (**b**). Hydrodynamic size and zeta potential information, determined by DLS (Ag NPs at 10 mg·L^−1^), are presented in (**c**) and (**d**) respectively. Data are mean ± SEM (*n* = 3), significant differences are represented as *p* < 0.1 (*) or *p* < 0.05 (**) following analysis with unpaired t-tests. Corresponding results obtained in artificial wastewater (AW) are presented in Supplementary Materials (Figure S7).

As Site 3 had particular characteristics compared to the other sites, especially a high content of chloride and sulfide, preliminary assays of aging were performed with CW3 and FW3 samples. The corresponding Ag NP toxicity and characterization related information post aging is presented in Figure 5.

Ag NPs showed a consistent toxicity pattern for four weeks in FW3 (*i.e.*, IC_50_ at 1 h close to 7.8, 7.8, 6.9 and 6 mg·L^−1^, RSE *ca*. 10%, at Week 0, 1, 2 and 4, respectively) before becoming less toxic by Week 8 (*i.e.*, IC_50_ at 1 h close to 12.6 ± 1.2 mg·L^−1^) (Figure 5a). Overall, comparable patterns were obtained with aging in AW (Figure S7). In CW3, aging effects were visible from Week 1, where IC_50_ values were twice the values derived with non-aged materials; then IC_50_ almost doubled again by the end of Week 8 (Figure 5a). Absorbance spectra showed changing patterns through the weeks compared to non-aged materials, especially in CW3, as no proper spectra were eventually visible in Week 8 (Figure 5b). In addition, general trends such as the increase in hydrodynamic size and the decrease of the zeta potential were also observed in CW3 with aging (Figure 5c,d). A decrease in the dissolution rates, especially in CWs (*i.e.*, from *ca.* 3% in Week 0 to 1% in Week 1 then below 1% in Week 8) was also observed with aging.

## 3. Discussion

### 3.1. Toxicity of Pristine Ag NPs in CWs and FWs

The adverse effect of Ag NPs to bacteria, although still mechanistically unclear, is commonly attributed to the released ions, which may exhibit greater toxicity by several orders of magnitude than their NP counterparts [29,30]. We previously reported IC_50_ values at 1 h of exposure close to 5 mg·L^−1^ for pristine Ag NM-300K NPs and 0.4 mg·L^−1^ for Ag ions in artificial wastewater (AW) simulating effluent using the same *P. putida* bioreporter used here [18]. The toxicity was shown to be mainly driven by the released ions, corroborating other literature [31]. The results obtained here in FWs between 6 and 9 mg·L^−1^ appear most of all directly comparable to those already presented in AW using the same method and material; they are consequently likely to be explained by the same impact of the released Ag ions.

The pristine Ag NPs were less toxic in CWs than FWs, with IC_50_ values at 1 h of exposure up to *ca.* 50 mg·L^−1^. This was associated with higher BOD, COD, TSS, total plate count, ammonia, chloride, sulfide loadings in CWs, attesting overall to the higher “complexity” of CWs compared to FWs. No evident aggregation of the freshly added NPs to the wastewaters was visible by UV-vis. No different dissolution rates of pristine NPs were evaluated in both matrices. However, significantly different hydrodynamic size and zeta potential values were measured in CWs compared to results in FWs, attesting to matrix driven effects on the NPs (*i.e.*, complexation) in CWs. Aggregated (*i.e.*, complexed) Ag NPs have been shown to display lower ion release and diminished bioavailability in complex matrices [24,32,33]. Ions themselves were shown to be susceptible to complexation in wastewaters [34,35]. Altogether, the reported data therefore support the hypothesis that aggregation and complexation of Ag NPs and related ions led to attenuated toxicity of Ag NPs to *P. putida* bioreporter in the most complex matrices, the CWs.

The acute toxicity (*i.e.*, IC_50_ values) of pristine Ag NPs to bacteria was reported in the mg·L^−1^ range in laboratory media [30,36]. Comparatively, similar and attenuated toxicity of Ag NPs were therefore reported here in FWs and CWs, respectively. Interestingly, both the microbial resilience [37,38] and sensitivity [39,40] were reported before in influent wastewaters testing Ag NPs, respectively, below and above 5 mg·L^−1^. The present work therefore further supports those previous studies.

Consequently, while demonstrating the workability of the proposed method for performing acute testing in real wastewaters the results from this work highlight further the importance of the matrix nature in determining fate and toxicity.

### 3.2. Comparative Toxicity of Aged Ag NPs

The aging impact in wastewaters on the eventual antibacterial properties of Ag NPs was reported here. Interestingly, Ag NPs showed consistent toxicity patterns in FWs despite aging. Comparable behaviors were obtained in AW. In both cases, this was related to steady hydrodynamic sizes and dissolution rates. Conversely in CWs, the increase in hydrodynamic size and zeta potential as well as the loss of typical spectra of absorbance and the decrease in dissolution (*i.e.*, traducing aggregation and complexation of NPs), then eventually in Ag NP toxicity, were characterized with aging.

Although reports on the speciation of aged Ag NPs are abundant [25,26,27], very little information exists on actual toxicity of aged Ag NPs, especially in real matrices. Ag NPs discharged to the wastewater stream were shown to be sulfidized to varying degrees and to be largely aggregated with biomass and biosolids [24,25,27]. Although there is clearly controversy on the risks and benefits of Ag NP containing consumer and healthcare products [41], the global impact of Ag NPs to WWTPs (*i.e.*, therefore their actual toxicity in CWs) may be limited at the present time [33]. Here, the toxicity based results reported in CWs with aged NPs may further corroborate these recent reports. However, our results in FWs suggest further attention should be given to impact of Ag NPs on environments downstream of WWTPs, especially as NPs have been already shown capable of escaping WWTPs [42].

Consequently, while piloting aging based assays we highlighted the divergent fate of the patterns of toxicity of Ag NPs in CWs and FWs (despite “conservative” conditions of aging). Clearly further assays using aged NPs from flume systems or full-scale WWTPs are needed.

### 3.3. Relevance and Future Work

*P. putida* is a common model microorganism in environmental science (e.g., water and soil); its sensitivity to NPs was previously reported [18,43,44] and its suitability for performing testing in real matrices was anticipated [28]. CWs, exhibiting BOD and TSS loadings *ca.* 200 mg·L^−1^ were representative of medium urban influent WWs. The characteristics of used FWs were in compliance with the European directive 91/271/EEC. Correlatively, the tested NPs are widely used representative materials from the European Commission, excluding Ag NM-300K NPs of being just a specific case study. The broad relevance of the approach selected is fully justified.

The behavior of Ag NPs has already been shown to be influenced by pH, ionic strength, salinity and presence of chloride or sulfide when studied separately in artificial conditions [34,45,46,47]. Considering FWs and CWs both collectively (*i.e.*, regardless of the site); *ca.* 75% of the observed variability overall was explained by the performed multivariate analysis (*i.e.*, based on a canonical correlation using key wastewater parameters constrained by the calculated IC_50_ of spiked NPs). On a wastewater type basis, the direct influence of the site or of key physico-chemical parameters to the eventual Ag NP toxicity cannot be drawn. It is possible that the dose used (up to 200 mg·L^−1^) may have exhausted the available sulfide and chloride limiting the potential impact of Ag NP. However, Kaegi *et al.* (2013) [27] demonstrated that Ag NPs were only sulfidized at 15% within 5 h in WWs, as also proposed elsewhere [24]. In addition, the sulfidization of Ag NPs was shown to be limited in oxic conditions [48]. Consequently, in the absence of aging, the actual impact of such sulfidization is uncertain here when considering data obtained after 1 h of exposure in oxic conditions. Meanwhile, although differences were evident between samples from the different sites they were probably too low overall to obtain a conclusive assessment of the direct effects of matrix parameters on the fate and toxicity observed. Studies in the literature discussing such aspects (*i.e.*, separately) have generally deployed concentrations or conditions which ranged several orders of magnitude therefore exacerbating, potentially, the effects observed when compared to conditions offered by real samples [34,45,46,47].

The literature discussing significant impact of NPs to WWTP processes refer to concentrations above the mg·L^−1^ range using pristine NPs [39,40]. Retention times of CWs may count from hours to weeks; we reported here further attenuated antibacterial properties of aged Ag NPs in CWs after only one week. Environmental concentrations of Ag NPs were recently proposed to be in the µg·L^−1^ range in wastewaters, in the µg·g^−1^ in biosolids and below the µg·L^−1^ in surface waters [49]. Consequently, and if this is the case, in the absence of massive release (voluntary or involuntary) overall information suggests a limited concern associated with aged NPs in wastewaters at the present time.

Based on a relevant environmental model bacterium, the methodology presented here has shown to be a rapid and an inexpensive solution for high throughput toxicity testing assays of NPs in real matrices. Additional work with various materials (e.g., type, size, shape) may be anticipated. The acute testing in AW, FWs and CWs with TiO_2_ NM-104 and ZnO NM-110 NPs did not result in clear toxicity patterns (data not shown). Complementary studies with the same bioreporter in different matrices or with alternative bioreporters in comparable matrices are of interest for the development of an array of broader applicability. In times where the lack of appropriate, simple and standardized procedures is largely stressed in nanoecotoxicology [11], such methodology should be diligently taken into account for performing acute testing of pristine and aged NPs. In addition, the reported method could be extended for applications using freshwaters or adapted to soil samples, opening further the scope of feasibility.

## 4. Materials and Methods

### 4.1. Materials

Representative Ag NPs (Ag NM-300K NPs, recently denominated JRCNM03002a) primary size *ca.* 15 nm, in suspension at 10% (w/v) in 4% (v/v) each of polyoxyethylene glycerol trioleate and polyoxyethylene (20) sorbitan mono-laurat) from the Organization for Economic Co-operation and Development (OECD) were obtained via the European Commission’s Joint Research Centre (Ispra, Italy) and characterized previously [18,50].

Real urban wastewater samples were collected from four distinct WWTPs (referred to as Site 1 to 4) in the central belt of Scotland. Both crude wastewater (CW: influent collected between the primary bar screen and the first clarifier) and final wastewater (FW: effluent collected after the last clarifier from where effluents are discharged to the water course) samples were obtained and tested for each site. All samples were used within 24 h following collection.

### 4.2. Methods

#### 4.2.1. Acute Testing Using Freshly Added Ag NPs

The switch-off *Pseudomonas putida* BS566::luxCDABE bioreporter in 96-well plate assay format was used as previously reported, with minor modifications [18]. Bacteria were pre-cultured overnight at 28 ± 2 °C under shaking conditions (140 rpm) in artificial wastewater (AW) then freshly diluted in order to reach a final concentration *per* well of 10^8^ CFU·mL^−1^. Stock suspensions of Ag NPs were freshly prepared at 222 mg·L^−1^ (*i.e.*, corresponding to 200 mg·L^−1^ final when used at 90% (v/v)) in collected wastewater samples prior to each experiment (*i.e.*, via weighting of NPs) then further serial diluted to give final tested concentrations of 0, 0.78, 1.56, 3.125, 6.25, 12.5, 25, 50, 100 mg·L^−1^. All wastewater samples were supplemented with D-glucose (0.5%, w/v) prior to use in order to ensure a consistent minimal amount of carbon source. Assays were conducted with 90 µL CW or FW (spiked with Ag NPs) mixed with 10 µL AW (containing the *P. putida* bioreporter) in black walled 96-well microtiter plates (Greiner bio-one, Germany). Monitoring of the emitted luminescence evolution was performed using a SpectraMax M5 reader (Molecular Devices, Sunnyvale, CA, USA) in a kinetic mode for 2 h at 28 ± 2 °C. Results were expressed in Relative Luminescence (% RLU) and plotted against time (min) for selected conditions. Ag NP toxicity was expressed as IC_50_ (mg·L^−1^) as derived at 1 h.

#### 4.2.2. Characterization of the Matrices

Before spiking with NPs, all wastewater samples were characterized for: BOD, as determined by a ROHASYS BOD robot (Rijen, the Netherlands) fitted with WTW Oxi 340i meter and corresponding CellOx 325 oxygen probe (samples incubated at 20 ± 1 °C for 5 days ± 4 h); COD, as determined by Hach^®^ test kits LCK314 and LCK114 read on a DR3800 spectrophotometer (Salford, UK); TSS, as determined by gravimetric analysis; and ammonia, as determined spectrophotometrically by a KONE auto-analyzer using the salicylate method. The total plate counts (performed on vegitone plate count agar with serial dilutions till 10^−7^ in NaCl 0.85% (w/v) and read after 48 h incubation at 37 °C) was also performed and the pH measured (HI 8424 pH meter, Hanna instruments Ltd, Leighton Buzzard, UK) for each sample prior to use. In addition, concentrations of chloride and sulfide were measured using a DR2000 spectrophotometer with dedicated kits (Hach^®^, Salford, UK) based on mercuric thiocyanate and methylene blue methods, respectively, following the manufacturers’ recommendations (*i.e.*, methods 8113 and 8131). Finally, the background amount of silver was determined by atomic absorption spectroscopy (AAS) using an AAnalyst 200 Spectrometer (Perkin Elmer, Waltham, MA, USA). Both unacidified and acidified (in 5% final nitric acid, v/v) wastewater samples were measured.

#### 4.2.3. Characterization of the Ag NPs

As described previously [18] Ag NP suspensions were characterized by UV-visible spectrophotometry (UV-vis) in clear disposable cuvettes using an Evolution 600 Spectrophotometer (Fisher Scientific Ltd, Loughborough, UK) and by dynamic light scattering (DLS) in clear disposable DTS1070 capillary cells using a Nano Zetasizer (Malvern Instruments Ltd, Malvern, UK). Concentrations of NPs as well as dissolution rates (in mass % terms) were assessed by AAS using an AAnalyst 200 Spectrometer in unacidified supernatants of ultra-centrifuged samples (30 min at *ca*. 50,000 g and 4 °C, Avanti Centrifuge J-26XP from Beckman Coulter (UK) Ltd, High Wycombe, UK) as also reported elsewhere [18,51]. The AAS apparatus was calibrated following the recommendations of the manufacturer using Ag pure single element standard at concentrations of 0.156, 0.312, 1.25, 2.5 and 5 mg·L^−1^.

#### 4.2.4. Impact of Aging

Acute testing and characterization assays (using freshly added Ag NPs, *i.e*., herein referred to as pristine NPs) were compared with aged Ag NPs in wastewaters. Stock suspensions of Ag NPs in CWs and FWs were stored in the dark at 4 °C for up to eight weeks and tested in Weeks 0, 1, 2, 4 and 8 following the aforementioned methods.

#### 4.2.5. Data Analyses

Data are mean ± standard error of the mean (SEM) from three to four independent experiments (each including four replicates *per* condition). IC_50_ values were derived by fitting a four parameter concentration-response model to the logarithm of the concentration using Prism package (GraphPad Software, La Jolla, CA, USA). Multivariate analysis (*i.e.*, canonical correlation analysis) was performed using Canoco5 considering BOD, COD, TSS, ammonia, chloride, sulfide, pH, plate count data as environmental variables constrained by the derived IC_50_ values as explanatory variables. Statistically significant differences between univariate results were tested using unpaired t-tests considering two-group cases at a time (*i.e.*, assessing a potential site effect or a potential wastewater type effect) with Prism (GraphPad Software, La Jolla, CA, USA).

## 5. Conclusions

This paper reports on the acute toxicity assessment of pristine and aged Ag NPs in real crude and final urban wastewaters (collected from four distinct WWTPs) using a bioluminescent switch-off *P. putida* bioreporter. Results were discussed in light of key physico-chemical parameters of the tested NPs and the spiked wastewaters. The main conclusions of the study are:

- Ag NPs exhibited ion based toxicity above the mg·L^−1^ range in all wastewater samples with toxicity patterns occurring at significantly lower concentrations in FWs compared to CWs;
- the impact of the wastewater composition on toxicity was driven by related BOD, COD, TSS, bacterial plate count, ammonia, chloride and sulfide loadings, which were all significantly more abundant in CWs;
- no significant site (*i.e.*, WWTP) effect was observed on Ag NP toxicity despite clear differences in the physico-chemical characteristics between FWs and CWs; and
- Ag NP toxicity decreased significantly with aging in CWs due to occurring aggregation and complexation phenomena, but not in FWs.

Not all bacteria are able to survive in real matrices (e.g., wastewaters), which emphasizes further the value of developing assays based on bespoke bioreporters originally isolated from such matrices, as the *P. putida* reported herein. In addition, the proposed microtiter plate format is easy to standardize and could also be performed as an array, which supports the current need of high throughput testing methods in nanoecotoxicology.

## Figures and Tables

**Table 1:** Physicochemical and microbiological parameters of used wastewaters. Data are mean ± SEM (n = 4).

Collection site	Site 1		Site 2		Site 3		Site 4	
Wastewater type	Crude	Final	Crude	Final	Crude	Final	Crude	Final
BOD (mg·L^−1^)	181 ± 13.7 ^a^	≤3	163.7 ± 11.7 ^a^	≤3	166.4 ± 25.7 ^a^	16.8 ± 3.2 ^b^	133 ± 23.6 ^a^	≤3
COD (mg·L^−1^)	393.4 ± 30.9 ^a^	23.8 ± 3.1	407.2 ± 40.2 ^a^	22 ± 2.7	402.8 ± 51.3 ^a^	58.2 ± 4.9 ^b^	342.5 ± 70.5 ^a^	18.2 ± 1.1
BOD/COD ratio	0.46 ± 0.03 ^a^	≤0.15	0.41 ± 0.03 ^a^	≤0.16	0.41 ± 0.01 ^a^	0.28 ± 0.03 ^b^	0.40 ± 0.02 ^a^	≤0.18
TSS (mg·L^−1^)	203.6 ± 23.9 ^a^	≤10	235.8 ± 31.3	N/A	224.2 ± 29.4 ^a^	28.2 ± 2.7	179.7 ± 57.9	N/A
Ammonia (mg·L^−1^)	15.8 ± 1.1 ^a^	0.63 ± 0.19	17.5 ± 1.6 ^a^	0.43 ± 0.14	18 ± 2.2	20.5 ± 2.6 ^b^	32.6 ± 10.5 ^a^	0.125 ± 0.075
pH	6.8 ± 0.16	7 ± 0.08	6.59 ± 0.26	6.50 ± 0.2	6.45 ± 0.09 ^a^	7.56 ± 0.06 ^b^	7.12 ± 0.04	6.94 ± 0.12
Total plate count (CFU·mL^−1^)	9.2 ± 5.2 × 10^6 a^	1.1 ± 0.7 × 10^4^	1.5 ± 0.3 × 10^6 a^	3.3 ± 1.3 × 10^3^	4.1 ± 0.4 × 10^6^ ^a^	7.83 ± 1.9 × 10^3^	6.4 ± 2.8 × 10^6^	1.4 ± 0.9 × 10^4^
Ag (mg·L^−1^)	<0.1 ^c^	<0.1 ^c^	<0.1 ^c^	<0.1 ^c^	<0.1 ^c^	<0.1 ^c^	<0.1 ^c^	<0.1 ^c^
Chloride (mg·L^−1^)	119.6 ± 21.3	71.6 ± 12.9	95.8 ± 7.81 ^a^	47.5 ± 1.65	253.9 ± 37.9 ^a,^ ^b^	148.2 ± 29.8 ^b^	79 ± 8.2 ^a^	50.5 ± 1.7
Sulfide (mg·L^−1^)	0.166 ± 0.03 ^a^	<0.010	0.362 ± 0.088 ^a^	<0.010	3.681 ± 1.2 ^a,^ ^b^	<0.010	0.272 ± 0.09 ^a^	<0.010

BOD (biological oxygen demand); COD (chemical oxygen demand); TSS (total suspended solids); pH (potential hydrogen); CFU (colony forming units); N/A corresponds to incomplete series of data; Symbols < indicate that parameter was consistently below the lower detection limit of the method or of the apparatus; ^a^ Data are significantly different between Crude and Final samples for the considered site (unpaired t-test, *p* < 0.05); ^b^ Considered information is significantly different to results with the same type of samples (Crude or Final) from all other sites (unpaired t-test, *p* < 0.05); ^c^ Tested with (and without) acidification (in 5% v/v final nitric acid).

## Supplementary Materials

The following are available online at http://www.mdpi.com/2079-4991/6/3/49/s1. Figure S1: Real time monitoring of Ag NP toxicity in wastewaters from Site 2. Figure S2: Real time monitoring of Ag NP toxicity in wastewaters from Site 1. Figure S3: Real time monitoring of Ag NP toxicity in wastewaters from Site 3. Figure S4: Real time monitoring of Ag NP toxicity in wastewaters from Site 4. Figure S5: Derived toxicity values at 0.5 and 2 h. Figure S6: Ag NP size distribution. Figure S7: Effects of aging in AW.
