# Supplementary material for: Toxicity Testing of Pristine and Aged Silver Nanoparticles in Real Wastewaters Using Bioluminescent Pseudomonas putida
Source: Nanomaterials (Basel). 2016 Mar 11;6(3):49. doi: 10.3390/nano6030049 (PMC5302522; doi:10.3390/nano6030049)
Supplement: Supplementary file 1 [file nanomaterials-06-00049-s001.pdf]

Supplementary Materials: Toxicity Testing of Pristine and Aged Silver Nanoparticles in Real Wastewaters Using Bioluminescent *Pseudomonas putida*

Florian Mallevre, Camille Alba, Craig Milne, Simon Gillespie, Teresa F. Fernandes and Thomas J. Aspray

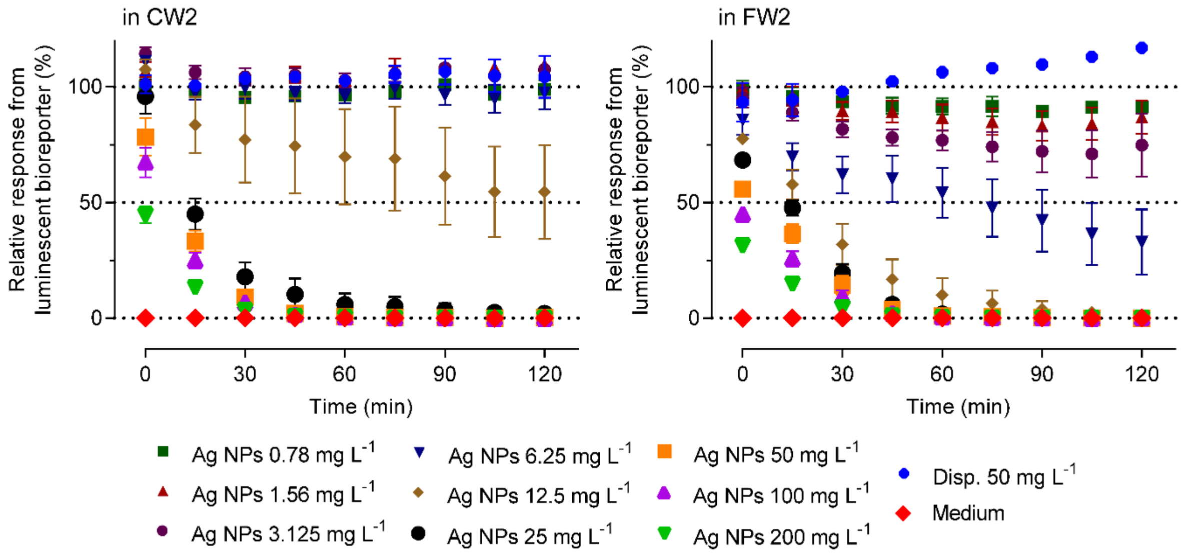

Figure S1. Real time monitoring of silver nanoparticle (Ag NP) toxicity in wastewaters from site 2. Relative luminescence output evolutions over time by *Pseudomonas putida* (*P. putida*) BS566::luxCDABE when challenged with 0–200 mg·L^{-1} of Ag NPs in crude or final wastewater samples from site 2 (CW2 and FW2, respectively) are shown. Background signal from used matrices and effect of Ag NP dispersant (at 50 mg·L^{-1}) are also proposed. Data are mean ± standard error of the mean (SEM) (n = 4).

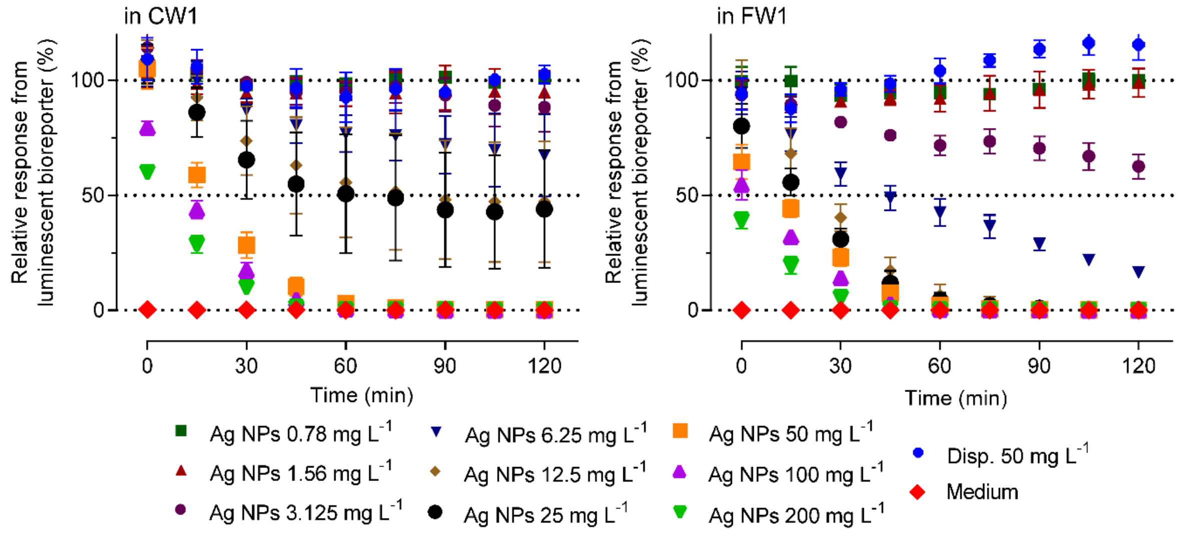

Figure S2. Real time monitoring of Ag NP toxicity in wastewaters from site 1. Relative luminescence output evolutions over time by *P. putida* BS566::luxCDABE when challenged with 0–200 mg·L^{-1} of Ag NPs in crude or final wastewater samples from site 1 (CW1 and FW1, respectively) are shown. Background signal from used matrices and effect of Ag NP dispersant (at 50 mg·L^{-1}) are also proposed. Data are mean ± SEM (n = 4).

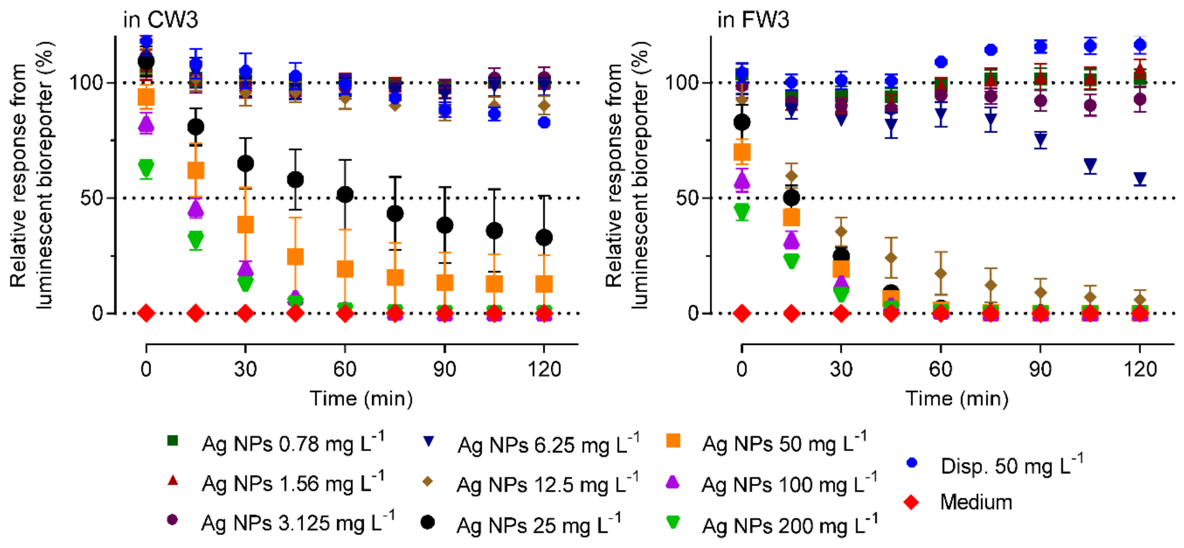

Figure S3. Real time monitoring of Ag NP toxicity in wastewaters from site 3. Relative luminescence output evolutions over time by *P. putida* BS566::luxCDABE when challenged with 0–200 mg·L^{-1} of Ag NPs in crude or final wastewater samples from site 3 (CW3 and FW3, respectively) are shown. Background signal from used matrices and effect of Ag NP dispersant (at 50 mg·L^{-1}) are also proposed. Data are mean ± SEM (n = 4).

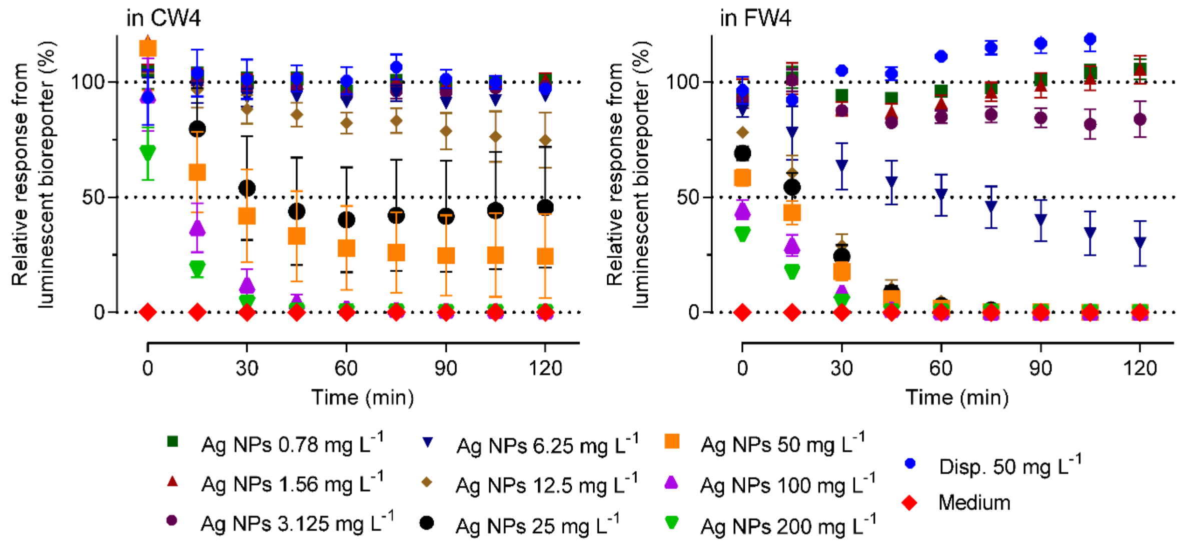

Figure S4. Real time monitoring of Ag NP toxicity in wastewaters from site 4. Relative luminescence output evolutions over time by *P. putida* BS566::luxCDABE when challenged with 0–200 mg·L^{-1} of Ag NPs in crude or final wastewater samples from site 4 (CW4 and FW4, respectively) are shown. Background signal from used matrices and effect of Ag NP dispersant (at 50 mg·L^{-1}) are also proposed. Data are mean ± SEM (n = 4).

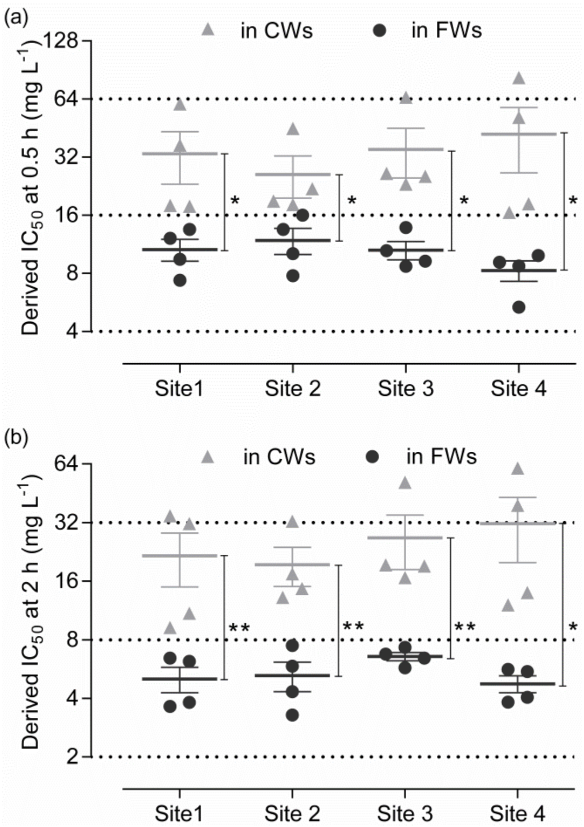

Figure S5. Derived toxicity values at 0.5 and 2 h. Toxicity results from light output reductions by *P. putida* BS566::luxCDABE when exposed to Ag NM-300K NPs in crude or final wastewaters (CWs and FWs, respectively) from four different wastewater treatment plants (WWTPs) (site 1 to 4) were plotted as (response) = *f*(log[Ag NPs]) for selected time points and IC_{50} values (half maximal inhibitory concentrations) were derived by fitting a four parameter concentration-response model. Calculated IC_{50} values at 0.5 and 2 h are shown in (**a**) and (**b**), respectively. Data are mean ± SEM ($n = 4$), significant differences by unpaired t-test are represented with $p < 0.1$ (*) or $p < 0.05$ (**).

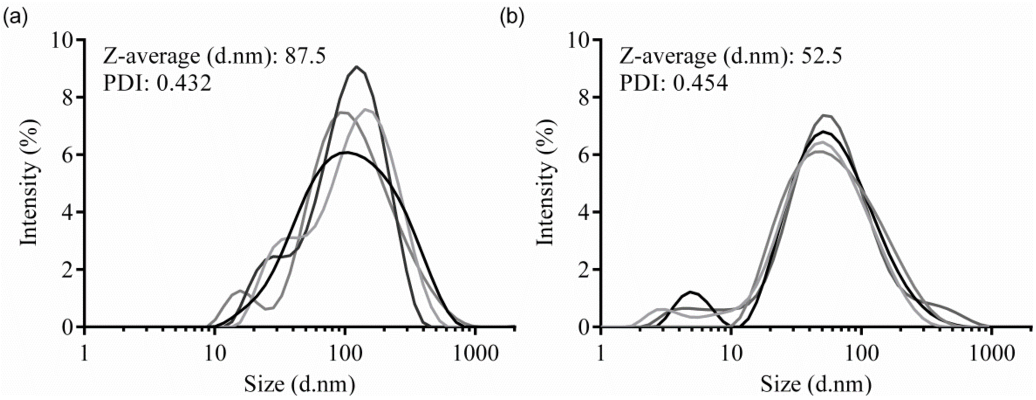

Figure S6. Ag NP size distribution. Examples of dynamic light scattering (DLS) output data (in intensity terms) with Ag NP spiked crude and final wastewaters (CWs and FWs) are shown in (**a**) and (**b**), respectively. Each graph presents four replicated measurements of the tested material (Ag NPs at 10 mg·L^{-1} in CWs or FWs) along with the corresponding Z-average or hydrodynamic size (as diameter, in nm) and polydispersity index (PDI).

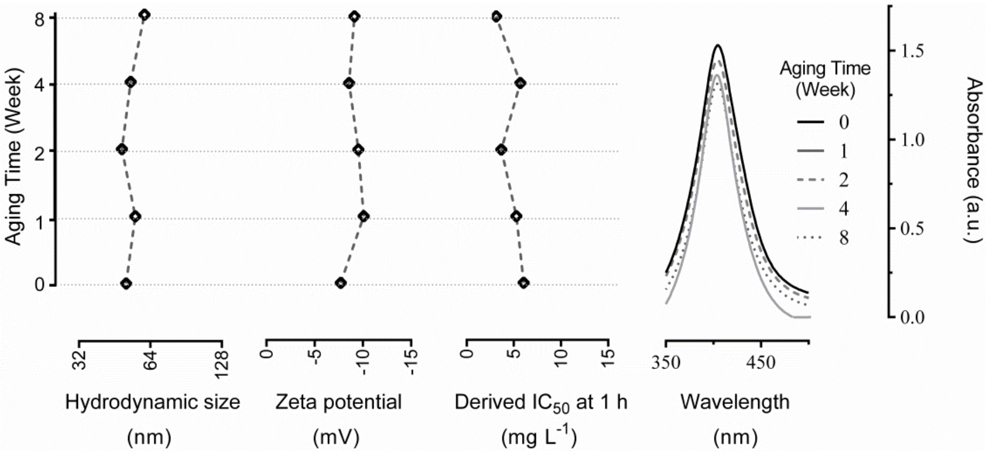

Figure S7. Effects of aging in artificial wastewater (AW). Fate and toxicity of Ag NPs were tested after 0, 1, 2, 4 and 8 weeks of aging in AW. Hydrodynamic size and zeta potential information (determined by DLS with a concentration of 10 mg·L^{-1}), derived IC_{50} values at 1 h (from ecotoxicity assays with concentrations of Ag NPs up to 200 mg·L^{-1}), and absorbance spectra (obtained by ultraviolet-visible (UV-vis) spectroscopy with a concentration of 10 mg·L^{-1}) are presented.

© 2016 by the authors; licensee MDPI, Basel, Switzerland. This article is an open access article distributed under the terms and conditions of the Creative Commons by Attribution (CC-BY) license (http://creativecommons.org/licenses/by/4.0/).
